# Implantable Cardioverter-defibrillators in Adult Congenital Heart Disease

**DOI:** 10.19102/icrm.2018.090601

**Published:** 2018-06-15

**Authors:** Merrill H. Stewart, Scott L. Macicek, Daniel P. Morin

**Affiliations:** ^1^Department of Cardiology, Ochsner Medical Center, New Orleans, LA, USA; ^2^Department of Pediatric Cardiology, Ochsner Medical Center, New Orleans, LA, USA; ^3^Ochsner Clinical School, University of Queensland School of Medicine, New Orleans, LA, USA

**Keywords:** Congenital heart defect, implantable cardioverter-defibrillator, sudden cardiac death, tetralogy of Fallot, transposition of the great arteries

## Abstract

With improved surgical techniques and medical therapies, many patients who are born with complex congenital heart defects are now living well into adulthood. As these patients age, an increasingly common cause of mortality is sudden cardiac death (SCD) from ventricular tachyarrhythmias. The implantable cardioverter-defibrillator (ICD) is a therapy with the ability to prevent some of these deaths; however, there are many diagnostic and technical challenges that remain in the congenital heart disease (CHD) population. We performed a literature review, searching PubMed for articles that examined the role of ICDs in CHD. We herein present the evidence for when to place an ICD in CHD patients, stratified by subtype as relevant. Then, we discuss the technical challenges and complications that are unique to this patient population. We conclude that, despite active work in the area, more research is needed given the small event rates and clinical variability within CHD populations.

## Introduction

Congenital heart disease (CHD), once only the domain of pediatricians, is fast becoming a chronic disease of adulthood. Improved surgical techniques have resulted in the prolonged survival of affected individuals, with more adults than children presently living with severe CHD.^1^ Current estimates suggest that more than 1.4 million adults and more than one million children in the United States are living with CHD.^2,3^ This paper will review indications for implantable cardioverter-defibrillator (ICD) implantation in CHD patients as well as technical considerations and issues surrounding the long-term management of such.

Late ventricular tachyarrhythmias (VTAs), or tachyarrhythmias occurring at a time later than 30 days after surgery, were recognized in CHD patients as early as in the 1970s. However, given the initial small number of patients, the issue of VTAs in adults with repaired CHD was not addressed on a systemic scale until the 1990s.^4,5^ In one study involving 6,024 patients with repaired CHD, sudden cardiac death (SCD) accounted for 22% of all deaths during a 45-year follow-up period. Among the common CHD patterns, tetralogy of Fallot (TOF), transposition of the great arteries (TGA) with a systemic right ventricle (RV), and univentricular heart (UVH) appear to be associated with the highest risk of SCD.^6^ For the purposes of this review, the scope of CHD will be limited to these distinct entities given their unique anatomic and clinical characteristics.

The ICD was developed in the early 1980s for use in adults to prevent SCD and became widely incorporated over the next two decades.^7^ The role of the ICD in CHD has taken more time to understand, especially given the relatively small patient population and CHD patients’ unique anatomic and physiologic considerations. The use of ICDs in CHD first began in the late 1980s, predominantly in survivors of sudden cardiac arrest; since then, primary prevention risk stratification and device utilization have evolved considerably.^8,9^

## Indications

By the late 1990s, the use of ICDs in CHD patients mirrored that in the larger adult market, moving into primary prevention for SCD.^10^ Indications regarding ICDs for secondary prevention of SCD in CHD patients were transferrable from the noncongenital adult population, but identifying those CHD patients who were the most appropriate candidates for primary prevention remained challenging. While many patients with CHD have an increased risk for arrhythmias, their varying pathophysiologies and natural histories makes the well-established risk factors for patients with other forms of cardiomyopathy not directly applicable in those with CHD. Current guidelines recommend ICD implantation in CHD patients who are survivors of cardiac arrest or who have symptomatic sustained VTA (a class Ib recommendation).^11^ The indications for primary prevention ICD implantation in CHD are not clear and there remains some discordance in the recommendations made in published guidelines, as shown in **Table 1**.^12^ In general, concerning features favoring ICD implantation include unexplained syncope, inducible monomorphic ventricular tachycardia (VT), systemic ventricular dysfunction, prolonged QRS duration, and heart failure symptoms.^11,12^ These criteria are based largely on nonrandomized case series sourced from the last 20 years, which have tried to identify CHD patients at the highest risk for SCD. A thorough understanding requires an evaluation of each specific subtype of CHD.

### Tetralogy of Fallot

TOF is the most common of the cyanotic and complex CHDs.^13^ It carries a significant risk of SCD, particularly increasing at the point beginning 20 years after repair, from 2% at 20 years to 4% at 25 years and 6% at 30 years, respectively.^14^ SCD represents approximately half of all late deaths (ie, deaths occurring at a time more than 30 days after operation) in patients with TOF.^15^ Many observational studies have investigated which TOF patients are at the highest risk and thus who might most benefit from prophylactic ICD implantation. For example, Gatzoulis et al. examined 793 TOF patients for a mean time of 21 years after repair.^16^ Of these, 33 developed monomorphic VT and 16 suffered SCD. As compared with patients who survived arrhythmia-free, the SCD group was noted to have an increased QRS duration (179 ms versus 146 ms; p < 0.0001) and an older age at the time of repair (17 years of age versus 7.5 years of age; p < 0.0001).^16^ A later study refined this QRS measurement to show that a duration of > 180 ms was most predictive of SCD and that RV hypertrophy increased SCD risk as well.^17^

Several other SCD risk factors have been identified among the TOF population, as shown in **Table 2**.^18^ The RV plays a key pathophysiologic role in VT late after TOF repair, with one study indicating that most patients with VT also have significant pulmonary regurgitation and/or RV outflow tract aneurysms.^19^ Valente et al. reviewed 873 patients with repaired TOF and an available cardiac magnetic resonance imaging scan as part of the International Multicenter TOF (INDICATOR) Registry, looking for a primary endpoint of death or sustained VT.^17^ They found that RV size did not correlate with their primary endpoint (unlike in other studies), but that a reduced RV ejection fraction (42% versus 49%; p = 0.004) did correlate with adverse outcomes. RV systolic pressure was also higher in those who reached the primary endpoint as compared with those who did not (64 mmHg versus 40 mmHg; p < 0.001).^17^

The left ventricle may also play a role: Khairy et al. showed that increased left ventricular end-diastolic pressure (LVEDP; 16 mmHg versus 10 mmHg, p = 0.001) correlated with the delivery of appropriate ICD therapy and that LVEDP was also inversely correlated with left ventricular ejection fraction (p = 0.0496).^20^ The study by Valente et al. additionally found an increased incidence of death and sustained VT in patients with decreased left ventricular ejection fraction (53% versus 58%; p = 0.01).^17^

Programmed ventricular stimulation (PVS), typically done at invasive electrophysiology study, has revealed that inducible VTA may correlate with future SCD and mortality. Event-free survival at 10 years in one study of 252 patients was 89% in those without inducible VTA versus only 62% in those with inducible VTA (p < 0.0001).^21^ Given the dire consequences of SCD in comparison with the lack of perfect specificity from this (or any) screening test, it is recommended that the data from PVS be considered in the overall decision-making process, rather than taken as an absolute indicator. Furthermore, it should be remembered that, though inducible VTA with PVS increases the risk of SCD, the absence of VTA on PVS does not automatically indicate an absence of SCD risk.

Cardiovascular magnetic resonance (CMR) imaging has emerged as a powerful tool in the evaluation and risk stratification of patients with CHD. CMR was used in TOF as early as 2006.^18^ At its most basic level, CMR offers an extremely accurate assessment of RV size and function to determine the prognostic metrics discussed above.^17,22^ Unique to CMR, though, is the ability to assess late gadolinium enhancement (LGE), a finding suggestive of myocardial fibrosis. Babu-Narayan et al. examined CMR in 92 patients with repaired TOF and found LGE present at the site of the RV outflow tract myomectomy in all patients and in other areas, such as the RV trabeculae, not associated with surgical manipulation.^18^ Greater LGE of the RV was associated with worse symptoms, RV dysfunction, and arrhythmia with symptoms and/or syncope.^18^

Identified risk factors for SCD and VT in repaired TOF help to explain and build upon the known pathophysiology of VT in this disease. As early as 1992, VT in TOF was mapped to the RV outflow tract. This is an area of surgical myomectomy in the typical TOF repair, and the VT in this area often has reentry as its mechanism.^23^ Outflow tract aneurysms after repair have been associated with SCD and VT and also could be a source of reentrant arrhythmias.^19^ This mechanism may explain why PVS performed at the time of electrophysiology study, which is more able to induce reentrant arrhythmias, has been shown to be of greater clinical utility in TOF than in TGA.^21^ Several markers of RV structural abnormalities and dysfunction have been shown to be related with SCD and VT, specifically QRS duration, RV systolic dysfunction, and RV ejection fraction. These risk factors could either indicate RV myocardial strain and thus a separate etiology of VT, or could be secondary markers of pulmonary insufficiency in the setting of more extensive outflow tract instrumentation and patch size (an already-mentioned risk factor for VT and SCD).

With respect to these various risk factors, in 2008, Khairy et al. attempted to devise a risk score for ICD discharges in TOF. They followed 121 patients with TOF who had an ICD for a mean of 3.7 years. After calculating actuarial annualized event rates for appropriate ICD discharges in primary prevention patients according to their clinical characteristics, they identified six risk factors and assigned each of them a point value on the basis of their relative risk as follows: prior palliative shunt (two points), inducible sustained VT (two points), QRS duration ≥ 180 ms (one point), ventriculotomy incision (two points), non-sustained VT (two points), and left ventricular end-diastolic pressure ≥ 12 mmHg (three points). Each one-point increase in the risk score was associated with a hazard ratio (HR) of 1.5 [95% confidence interval (CI): 1.2–1.8; p = 0.0003].^20^

### Transposition of the great arteries

TGA with repair involving an atrial switch (typically via either the Mustard or Senning procedure) is a long-recognized risk factor for late SCD. In one study of 534 patients conducted between 1963 and 1994, there were 77 late deaths (16%) and, of these, 50% (n = 33) were sudden in nature.^24^ In contrast with TOF, there is much less of a delay in the occurrence of SCD in patients with TGA, with incidences of 4% at 10 years after surgery and 9% at 20 years after surgery, respectively.^14^ Risk factors associated with SCD in this population also have been examined. For example, Kammeraad et al. considered 47 patients with repaired TGA and SCD and matched them 1:2 with repaired TGA patients without SCD. They found that those with SCD had an increased prevalence of atrial fibrillation/atrial flutter.^25^ Unfortunately, the pathophysiologic role of these atrial arrhythmias remains unclear, as RV failure can cause increased atrial arrhythmias and RV dysfunction alone is a risk factor for ventricular arrhythmia. In addition to RV failure, the atrial switch procedure creates an arrhythmogenic substrate with multiple atrial scar lines, areas of slowed conduction, and potential reentrant pathways.^26^

Kammeraad et al. also demonstrated another unique twist in the TGA population: 34 of the 47 (81%) patients with repaired TGA and SCD died during physical exertion.^25^ The nature of the atrial switch operation with flow-limiting baffles could make cardiac output augmentation during exercise difficult. In addition, the often-failing systemic RV could be ill-equipped to meet the needs of a rapid increase in cardiac demand. This mismatch may be a setup for ischemia-induced arrhythmia, even in the absence of obstructive coronary disease. Another theory is that exercise increases the likelihood of rapid atrial arrhythmias being able to conduct to the ventricle in a 1:1 fashion, thus inducing ventricular tachyarrhythmias.^27^

As in patients with TOF, CMR has been used in those with TGA to precisely measure RV size and function. CMR has also been able to find LGE in the systemic RVs of individuals with TGA who have undergone an atrial switch procedure. In a small study of 36 patients, myocardial fibrosis as measured by LGE negatively correlated with RV ejection fraction. LGE was also associated with an increased incidence of syncope or symptomatic arrhythmia (nine of 22 versus one of 14; p = 0.03).^28^ In another prospective study involving 55 patients with TGA who had undergone an atrial switch procedure, LGE was present in 31 (56%) of patients. Patients with LGE had an increased incidence of atrial tachyarrhythmia (15 of 31 versus four of 24; p = 0.022) and ventricular tachyarrhythmia (six of 31 versus none of 24; p = 0.03), with an insignificantly higher risk of death (three of 31 versus none of 24; p = 0.248).^29^

Khairy et al.^30^ followed 37 patients with repaired TGA and an ICD, 62% of whom received device placement for primary prevention and 38% of whom received device placement for secondary prevention. They found that, unlike in TOF, PVS played no role in predicting ICD discharge. As discussed, this is understandable due to the lack of ventricular myomectomy in an atrial switch procedure (as opposed to in TOF repair) and thus the presence of less of a substrate for reentrant ventricular arrhythmia. In concordance with Kammeraad et al., Khairy et al. often found instances of atrial arrhythmias triggering ventricular arrhythmias that resulted in ICD discharge. The higher rate of atrial arrhythmias may be driven either by RV failure or by increased atrial scarring caused by TGA repair in comparison with that in TOF repair. They also found a very low annual rate of appropriate ICD shocks for patients who received their ICD for primary prevention versus secondary prevention (0.5% versus 6.0%; p = 0.036), demonstrating that less-than-ideal risk stratification was available for the primary prevention cohort.^25,30^

Whether RV failure results in atrial arrhythmias or whether atrial arrhythmias contribute to RV failure is unknown. However, what is known is that these atrial arrhythmias play a much more prominent role in the morbidity and mortality of TGA with atrial switch than in repaired TOF. In addition to tachyarrhythmias, sinus bradyarrhythmias are also an issue. Likely due to the different atrial incisions made, the Mustard procedure has been associated with a greater loss of sinus rhythm than the Senning procedure at five-year follow-up (64% versus 33%, p < 0.001).^31^ Indeed, one early study of 123 patients actually identified two deaths that occurred as a result of sinus node failure, and atrioventricular node dysfunction has also been identified in TGA patients.^32,33^ Thus, while it was thought that the majority of instances of SCD were the result of tachyarrhythmias, it also is possible that severe bradyarrhythmias may be contributing to SCD in this group.

### Univentricular heart

Because the concept of “single-ventricle physiology” covers a wide variety of different congenital abnormalities, generalization with respect to primary-prevention ICD indications is more difficult. These patients typically suffer more from atrial arrhythmias (as a result of right atrial pressure overload) than from ventricular ones. In one study of 261 patients who underwent Fontan repair, there was a 9% risk of SCD at 20 years postoperation noted, presumed to be arrhythmic in nature. However, there were no identifying risk factors in this group that would enable screening for primary prevention.^34^

## Guidelines

As discussed above, the combination of small patient populations, overall low event rates, and varying anatomy makes it difficult to identify CHD patients who are at high risk for SCD. The 2015 European Society of Cardiology (ESC) guidelines attempted to address these concerns with an expanded review of ICDs in CHD. For primary prevention, they gave a class I recommendation for ICD use in those with CHD, biventricular physiology, a systemic ejection fraction of < 35%, and heart failure symptoms. This was based on several large series and was encouraged by the reproducibility present in the general adult population.^10,35,36^ Beyond this, class IIa recommendations for primary prevention ICDs in CHD patients include (1) in the presence of unexplained syncope with a positive PVS or ventricular dysfunction; or (2) in individuals with TOF with additional risk factors such as left ventricular dysfunction, nonsustained VT, QRS duration > 180 ms, or inducible sustained VTA on PVS.^11^ These class IIa recommendations are largely based on the handful of series discussed in the TOF section above.^20,21^ Further, class IIb recommendations include ICD therapy in the setting of systemic right RV dysfunction with additional risk factors such as nonsustained VT, aortic regurgitation, or heart failure symptoms.

Notably absent from these guidelines are any specific risk factors for non-TOF CHD. This is important, given that many of the TOF risk factors have specifically been found not to be associated with SCD in other forms of CHD, such as surgically repaired TGA.^30^ Indeed, the guidelines fail to capture a large portion of those individuals who may benefit from ICD therapy. In a review of 25,790 CHD patients from a multicenter registry, Vehmeijer et al. identified 157 CHD patients who suffered SCD, among whom only 35% had a preexisting class I, IIa, or IIb recommendation for an ICD according to the ESC, thus suggesting that the majority of SCD cases within the CHD population would not meet current guidelines.^37^ An expert consensus statement released in 2014 by the Pediatric and Congenital Electrophysiology Society (PACES) and the Heart Rhythm Society (HRS) was similar in scope to the ESC guidelines, with the exceptions of including the presence of RV scar as a risk factor for TOF patients and of removing the requirement of heart failure symptoms in the setting of systemic ventricular dysfunction.^12^ In the review by Vehmeijer et al., the PACES/HRS guidelines did not fare much better, with only 41% of the patients with SCD also having an indication for ICD.^37^

While we have identified risk factors for SCD in patients with TOF, more analyses with large pools of data are needed to establish risk factors in the non-TOF CHD population. Walsh focused on risk stratification in a 2014 review of several types of CHD.^38^ For TGA, in addition to the guidelines-stated indications, he found the occurrence of atrial tachycardia and longer duration since surgery to be associated with an increased risk of SCD. Thus, these factors could be used when determining whether to implant a primary-prevention ICD in a TGA population. In patients with UVH, they identified the same risk factors as those in TGA (ie, atrial tachycardia and duration from follow-up) as well as certain indications already listed in published guidelines, such as syncope and depressed systemic ventricular function. Older Fontan techniques including the native right atrium in the Fontan circuit were also associated with increased SCD, likely due to increased atrial overload driving atrial arrhythmias.^38^

At this time, further risk stratification methods are being developed. For example, in another investigation from Vehmeijer et al. examining the same large database of 25,790 patients, the authors found that a fragmented QRS—that is, a QRS complex with one or more noncontinuous deflections in two contiguous leads—was associated with SCD. In this series, 51% of SCD patients had a fragmented QRS versus 34% of matched controls (odds ratio: 2.0; p = 0.003).^39^ While predictive, even this new marker fares only slightly better than already existing guidelines and thus must be included in the overall clinical picture in conjunction with other items of note.

## Technical considerations

The CHD patient population presents a unique spectrum of procedural concerns when it comes to ICD implantation. The first ICDs were implanted with epicardial patches surgically placed onto the heart, requiring a sternotomy. While the presence of an ICD in such cases was beneficial to those with recurrent malignant arrhythmias, there was substantial morbidity and mortality associated with the implant procedure and the subsequent presence of the epicardial patches. Then, in the early 1990s, transvenous leads were introduced, which markedly diminished the need for a surgical approach. The transvenous implantation technique quickly showed a significant reduction in mortality, driven by a decrease in procedural complications.^40^ Furthermore, in addition to decreased surgical mortality, transvenous leads have been shown to have better longevity and improved defibrillation efficacy.^41^ As a result of these advantages, despite their unusual anatomy, transvenous leads can be used in up to 97% of pediatric and adult CHD patients.^42^

Unfortunately, some CHD patients do not have typical venous conduits through which ICD leads may be placed. If such conduits do exist, there sometimes is particular concern for causing lead-induced venous occlusion or lead-related complications involving atrial baffles or venous grafts. In the absolute absence of venous access to the heart, such as following an extracardiac Fontan repair, various innovative strategies have been successful. In one series of eight patients, sternotomies were performed, after which, typically, transvenous defibrillation coils were placed directly into the pericardium or implanted within the RV endocardium via the right atrial wall approach.^43^ In another series of seven patients, pericardial leads were placed via a small subxiphoid incision, thus eliminating the sternotomy.^44^ Of note, another series demonstrated superior defibrillation thresholds following implantation with the subxiphoid approach when the defibrillation lead was placed anteriorly rather than posteriorly.^45^ An anterior approach actually resulted in a larger cardioversion energy field, as the lead in this case is able to cover more of the superior border of the heart.

Another concern with intracardiac device use in patients with CHD is the possibility of systemic embolization in the presence of an intracardiac shunt. Khairy et al. retrospectively examined a total of 202 patients with intracardiac shunts, 64 of whom had transvenous leads, 56 of whom had epicardial leads, and 82 of whom did not have any cardiac device. In a multivariable regression analysis, they found transvenous leads were associated with increased systemic thromboembolism risk (HR: 2.6; p = 0.027).^46^ Due to this risk, it is not recommended that transvenous leads be placed in patients with significant intracardiac shunts.^12^ When and if intracardiac leads are placed in patients with shunts, chronic systemic anticoagulation should be considered.

Given the potential for atrial arrhythmias in many CHD patients, some have advocated for the placement of ICDs with an atrial lead to allow for arrhythmia discernment. However, in a 2008 review of 168 pediatric and young adult patients, including 44 with structural CHD, who had either a dual-chamber or single-chamber cardiac device, there was no difference in the rate of inappropriate versus appropriate ICD therapy.^47^ Therefore, keeping in mind the added complexity of device extraction in these patients, it may be preferable to select a single-chamber device.

For those patients in whom transvenous lead implantation is possible, there may be additional factors to consider. In the presence of an atrial baffle, ICD leads traversing that space can potentiate stenosis of the baffle. Ultimately, if stenting is required, the ICD leads are extracted prior to stenting to avoid pinning the lead against the vessel wall. In addition, the presence of a lead in a stented location imparts a risk of incomplete expansion of the stent.^48^ When a baffle exists that eventually could become stenotic, it sometimes is preferable to prophylactically stent the baffle prior to ICD lead insertion.

Finally, in some situations, transvenous routes near the heart can be used to avoid pericardial lead placement. For example, one patient with a partial Fontan repair that routed the superior vena cava into the pulmonary artery had his transvenous coil placed in the azygous vein and had an anterior subcutaneous array added to allow the shock vector to subtend the heart.^49^

### Subcutaneous devices

**Figure 1:**
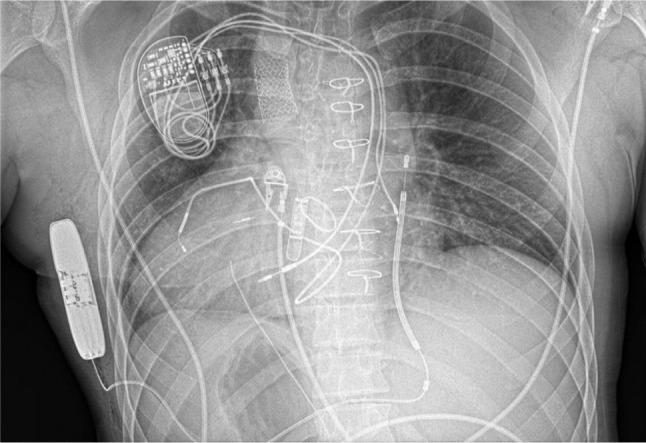
A patient with dextrocardia, atrial septal defect, ventricular septal defect, coarctation of the aorta, and aortic stenosis following mechanical aortic valve implantation with postsurgical complete heart block and subsequent dual chamber biventricular permanent pacemaker implantation. The patient later required an ICD for sustained symptomatic VTA seen on an implantable loop recorder. A right-sided subcutaneous device was chosen given the complexity of existing intravascular hardware, anatomic malalignment concerning for poor transvenous shock vector, and existing venous occlusions.

While subcutaneous arrays and leads have long been used in pediatric patients, ICDs specifically engineered for subcutaneous placement (S-ICDs) present an additional tool for use in CHD patients.^50^ However, the current iteration of the device has inherent limitations, with no capability for longstanding pacing for bradycardia, no ability for cardiac resynchronization therapy, and no antitachycardia pacing (ATP) option for the treatment of monomorphic VT. Nonetheless, in the absence of the need for these features, the S-ICD can be an attractive choice, especially given its lack of endovascular hardware **(Figure 1)**. The device initially had problems with T-wave oversensing and inappropriate shocks, which can be of particular concern considering the atypical surface electrocardiograms (ECGs) of some CHD patients with bundle branch block and/or atypical T-wave morphology.^51,52^ To prevent inappropriate shocks, it is imperative to preoperatively screen patients with surface electrode mapping. The screening process mimics an S-ICD tachycardia detection algorithm by measuring the surface ECG. The T-wave amplitude must be less than the QRS amplitude by a certain ratio at various times post-QRS. In one series of 30 adult patients with CHD, 86% were eligible for the S-ICD on the basis of surface mapping.^53^ While the true magnitude of inappropriate therapy in S-ICD use is unclear, in one study of 21 adult CHD patients with S-ICDs, there was only one instance of inappropriate discharge for T-wave oversensing over 14 months of follow-up.^54^ In another study of eight patients with CHD (seven adults and one child) followed for a median of 2.3 years, there were no inappropriate therapies administered during the entirety of follow-up.^55^ Anecdotally, other challenges to S-ICD implantation include patients with dextrocardia, for whom the implantation technique must be mirror-imaged. In addition, patients who have undergone one or more sternotomies may have significant chest wall deformities that make tunneling the lead along the sternum a more challenging task.

## Complications

The most common ICD-related complication encountered in the CHD population is inappropriate shocks for arrhythmias that are not life-threatening. This problem is not unique to these patients, however. For example, inappropriate shocks occurred in 83 (11%) of the 719 patients randomized to receive ICDs in the Multicenter Automatic Defibrillator Implantation Trial (MADIT)-II study that examined the role of ICD use postmyocardial infarction with reduced ejection fraction.^56^ What is different among CHD patients is the frequency of inappropriate shocks. For example, a 2016 meta-analysis of 16 studies found an inappropriate shock rate of 25% over 3.7 years among 518 patients with CHD.^57^ Several etiologies are likely to explain this high incidence of inappropriate therapy. Given the younger average age of this group, there is a higher likelihood of sinus tachycardia falling within the heart rate range of a device’s programmed tachycardia zone. There is also an increased incidence of atrial arrhythmias, either correlated with systemic RV overload or incision-related scars after surgical repair and/or cardiopulmonary bypass cannulation. Lastly, as many of these devices were placed in childhood, over time, there is a higher incidence of lead fracture, resulting in inappropriate discharges.^10,58,59^

Fortunately, the frequency of inappropriate ICD therapy can be partially mitigated via improved device programming. For example, a high baseline inappropriate therapy rate was noted in a non-CHD patient population in the MADIT-Reduce Inappropriate Therapy (MADIT-RIT) study, which randomized 1,500 patients with a primary prevention ICD to one of three different programming strategies. By increasing the lower limit of therapy from 170 beats per minute (bpm) to 20 bpm, the rate of inappropriate therapy was reduced from 20% to 4%, with an additional benefit of reduced mortality in the group whose devices were programmed to this higher VTA detection rate.^60^ Similar strategies have been applied in the CHD population. Techniques such as increasing the rate of therapy zones, prolonging detection times, allowing an increased number of ATP attempts, and ATP occurring during charging in the ventricular fibrillation zone are all strategies that have been found to be useful in CHD patients.^61,62^ An additional question that remains controversial with respect to the CHD population is the need for defibrillation threshold (DFT) testing at the time of implantation. Certain patients in whom the ICD lead position and can are not standard may warrant DFT testing at the time of implant.^63^ These nonstandard vectors include epicardial patch-to-can, subcutaneous array-to-can, and transvenous lead-in-subpulmonic left ventricle arrangements. As these vectors may not subtend the majority of the ventricular myocardium, defibrillation efficacy may be suboptimal. Preprocedural planning should include an evaluation of the need for DFT testing, particularly in those patients for whom a nontraditional implant approach is being utilized.

The second most common complication in the CHD population is lead failure. Lead failure (either fracture or insulation breach) can result in inappropriate discharges. It also can result in the need for placement of additional hardware and/or lead extraction with potential procedural morbidity and/or mortality. A large retrospective series of 1,007 pacemaker leads in 497 pediatric patients found a lead failure rate of 21% at a median follow-up time of 6.2 years. In a multivariable analysis, younger age at the time of implant (< 12 years) was associated with an increased risk of lead failure (HR: 2.70, 95% CI: 1.72–4.25), as was structural CHD (HR: 1.76, 95% CI: 1.16–2.67).^64^ A later review focusing only on ICDs found a similarly high rate of lead failure in children and in those with CHD, with an incidence of 5.6% per year (95% CI: 3.4–7.8).^59^ These numbers are significantly higher than those in the general adult ICD population, which carries an incidence of lead failure of only 0.29% to 0.45% per year.^65^ Given this higher rate of lead failure, proper patient selection and risk stratification are all the more important to focus on so as to avoid unnecessary morbidity and mortality.

## Extraction

The indications for ICD placement in those with CHD often arise early in life. With improving life expectancy as a result of surgical and medical therapy advances, many CHD patients require multiple device exchanges throughout their lifespan. As multiple generator changes are performed, infection risk rises, and, as leads age, a higher potential for lead damage exists. In addition, pediatric patients’ growth following implantation may cause lead malposition and/or dislodgement. When leads fail, often the decision is made to extract the failed leads, even in the absence of infection, in order to avoid an accumulation of endovascular hardware. Several series of such extraction procedures have been published, with a complication rate of 4% to 5% and no procedural mortality reported to date.^58,66,67^

## Conclusions

Given the improvements in surgical and medical therapy, patients with previously fatal complex congenital heart abnormalities are now living long into adulthood. With this increased survival, these patients’ long-term risk of SCD is becoming more apparent, and ICDs have become a therapeutic modality commonly used to mitigate this risk. Unfortunately, given the unique and variable nature of CHD, the same indications used in the general adult population cannot be universally applied in CHD patients. As such, the need remains to clearly identify those who are most at-risk of SCD in order to prevent avoidable deaths. CHD patients also have unique anatomic considerations that can make device implantation and extraction challenging. With time and greater experience, we hope that ICD therapy in CHD will continue to improve and that more lives may be saved with fewer complications.

## Figures and Tables

**Table 1:** Primary Prevention ICD Indications Stratified by Society

Class	2014 PACES/HRS Consensus Statement^12^	2015 ESC Guidelines^11^
Class I	ICD therapy is indicated in adults with CHD and a systemic left ventricular EF < 35%, biventricular physiology, and NYHA functional class II or III symptoms	ICD therapy is recommended in adults with CHD and a systemic left ventricular EF of < 35%, biventricular physiology, symptomatic heart failure despite optimal medical treatment, and NYHA functional class II or III symptoms
Class IIa	ICD therapy is reasonable in selected adults with TOF and multiple risk factors for sudden cardiac death, such as left ventricular systolic or diastolic dysfunction, nonsustained VT, QRS duration > 180 ms, extensive right ventricular scarring, or inducible sustained VT at electrophysiologic study	ICD implantation should be considered in selected patients with TOF and multiple risk factors for SCD, including left ventricular dysfunction, nonsustained VT, QRS duration > 180 ms, or inducible sustained VT on programmed ventricular stimulation
		ICD implantation should be considered in patients with CHD with syncope of unknown origin in the presence of either advanced ventricular dysfunction or inducible sustained VT, or ventricular fibrillation on programmed ventricular stimulation
Class IIb	ICD therapy may be reasonable in adults with a single or systemic right ventricular EF of < 35%, particularly in the presence of additional risk factors such as complex ventricular arrhythmias, unexplained syncope, NYHA functional class II or III symptoms, QRS duration > 140 ms, or severe systemic atrioventricular valve regurgitation	ICD therapy may be considered in patients with advanced single or systemic right ventricular dysfunction in the presence of other risk factors such as nonsustained VT, NYHA functional class II or III, or severe systemic atrioventricular valve regurgitation
	ICD therapy may be considered in adults with CHD and a systemic ventricular EF < 35% in the absence of overt symptoms (NYHA class I) or other known risk factors	
	ICD therapy may be considered in adults with CHD and syncope of unknown origin with hemodynamically significant sustained VT or ventricular fibrillation inducible at electrophysiologic study	
	ICD therapy may be considered in nonhospitalized adults with CHD awaiting heart transplantation	
	ICD therapy may be considered in adults with syncope and moderate or complex CHD in whom there is a high clinical suspicion of ventricular arrhythmia and in whom thorough invasive and noninvasive investigations have failed to define a cause	

ICD: implantable cardioverter-defibrillator; PACES/HRS: Pediatric and Congenital Electrophysiology Society/Heart Rhythm Society; ESC: European Society of Cardiology; CHD: congenital heart disease; EF: ejection fraction; NYHA: New York Heart Association; TOF: tetralogy of Fallot; VT: ventricular tachycardia; SCD: sudden cardiac death.

**Table 2:** Risk Factors for SCD in Patients with TOF

RV enlargement and dysfunction^16^
Older age at the time of repair^16^
Atrial arrhythmias^17^
QRS duration > 180 ms^17^
RV fibrosis on magnetic resonance imaging^18^
Elevated LV filling pressures^20^
Inducible VTA with programmed ventricular stimulation^21^
Symptomatic VTA^36^

SCD: sudden cardiac death; TOF: tetralogy of Fallot; VTA: ventricular tachyarrhythmia; RV: right ventricular; LV: left ventricular.
